# Transport Mechanisms and Dielectric Features of Mg-Doped ZnO Nanocrystals for Device Applications

**DOI:** 10.3390/ma15062265

**Published:** 2022-03-18

**Authors:** Chayma Abed, Amira Ben Gouider Trabelsi, Fatemah. H. Alkallas, Susana Fernandez, Habib Elhouichet

**Affiliations:** 1 Physics Department, Faculty of Sciences of Tunis, University of Tunis El Manar, Tunis 2092, Tunisia; 2 Department of Physics, College of Science, Princess Nourah Bint Abdulrahman University, P.O. Box 84428, Riyadh 11671, Saudi Arabia; 3 Renewable Energy Department, CIEMAT, 28040 Madrid, Spain; 4 Physics Department, College of Sciences, University of Bisha, P.O. Box 551, Bisha 61922, Saudi Arabia; 5 Laboratory of Characterizations, Applications and Modelisation of Materials LR18ES08, Sciences Faculty of Tunis, University of Tunis El Manar, Tunis 2092, Tunisia

**Keywords:** ZnO nanocrystals, Mg doping, polaron hopping, high-dielectric constant, modulus

## Abstract

Magnesium-doped zinc oxide “ZnO:Mg” nanocrystals (NCs) were fabricated using a sol gel method. The Mg concentration impact on the structural, morphological, electrical, and dielectric characteristics of ZnO:Mg NCs were inspected. X-ray diffraction (XRD) patterns display the hexagonal wurtzite structure without any additional phase. TEM images revealed the nanometric size of the particles with a spherical-like shape. The electrical conductivity of the ZnO NCs, thermally activated, was found to be dependent on the Mg content. The impedance spectra were represented via a corresponding circuit formed by a resistor and constant phase element (CPE). A non-Debye type relaxation was located through the analyses of the complex impedance. The conductivity diminished with the incorporation of the Mg element. The AC conductivity is reduced by raising the temperature. Its plot obeys the Arrhenius law demonstrating a single activation energy during the conduction process. The complex impedance highlighted the existence of a Debye-type dielectric dispersion. The various ZnO:Mg samples demonstrate high values of dielectric constant with small dielectric losses for both medium and high-frequency regions. Interestingly, the Mg doping with 3% content exhibits colossal dielectric constant (more than 2 × 10^4^) over wide temperature and frequency ranges, with Debye-like relaxation. The study of the electrical modulus versus the frequency and at different temperatures confirms the non-Debye relaxation. The obtained results reveal the importance of the ZnO:Mg NCs for device applications. This encourages their application in energy storage.

## 1. Introduction

Zinc oxide (ZnO) has been the subject of rapid development in the past decades in order to meet different research requirements. Essentially, its low-cost eco-friendly nature is behind its success. This explains the significant demand of ZnO material in several technological fields such as: solar cells [1], gas sensing [2], and light-emitting devices [3]. Indeed, ZnO material owns a wide band gap energy (≈3.37 eV) as well as a broad extinction binding energy (≈60 meV) at room temperature [4,5]. It is also distinguished with its notable dielectric characteristics, particularly, an elevated dielectric constant and a minor dielectric loss [6].

In this regard, the dielectric properties of ZnO are extremely attractive to researchers. Previous studies reveal the importance of developing materials with a high dielectric constant that ensures better energy storage in devices. Such materials have been widely applied to modern wireless communication technology [7] and electronic components such as capacitors. Similarly, fabricating material with low dielectric loss guarantees proper energy storage. In fact, low dielectric loss is required for non-linear optics domains, particularly at elevated frequencies [8].

ZnO materials have been enormously improved when associated with the appropriate oxides [9,10,11], 2D materials [12], and/or simple doping [13]. Herein, the properties of ZnO become highly sensitive to the nature of the dopant element and its concentration [14,15]. Its electrical properties depend on the excess of zinc at the interstitial position [16] and are affected essentially by the dopant element, such as, Fe [17], Al [18], and Sn [19]. However, its dielectric features drastically change with an element incorporated into its lattice [20,21,22]. Particularly, Mg was used to improve both the dielectric properties and the electrical conduction of ZnO. Indeed, the Zn substitution with the Mg element does not affect the lattice constants [23]. This is due to the high similarity between both elements, where their ionic radius is close to each other (i.e., Mg^2+^ = 0.057 and Zn^2+^ = 0.060). This results in a decrease in the defect’s density in the ZnO lattice through a specific amount of Mg doping which ameliorates the electrical and dielectric properties of ZnO.

Deep investigations are still needed into the structural, transport, dielectric, and modulus properties of ZnO NCs-doped Mg via a low-cost technique. Earlier studies confirm the encouraging performances of the electrical and dielectric properties of ZnO:Mg in numerous applications. O. Hafef et al. [24] demonstrated the decrease in AC conductivity with increasing MgO concentration in ZnO/MgO composites. This has been assigned to the sintering technique where ions scattering form deep donor levels in the band gap. However, other studies exhibited the diminution of the AC conductivity of the material for low Mg-doping amounts [25], while high-doping levels elevate the AC conductivity of the ZnO matrix [26]. Justin Raj et al. [27] succeeded in synthesizing ZnMgO ceramics with a colossal dielectric constant that emerged from grain defects and their boundary. This reduces the conductivity relaxation due to the adsorbed water molecules. Indeed, the adsorbed moisture in the porous structure of ZnO ceramics codoped with Li and Mg contribute to the anomalous dielectric constant [28]. A similar tendency of the dielectric constant was reported for porous ZnO ceramics treated at high pressure. Here, the changes between grains and their boundary resistance was owed to a relaxation mechanism of the Maxwell–Wagner type that causes such behavior [29].

In our previous investigations, we inspected the opto-structural properties of ZnO:Mg nanocrystals (NCs) [23]. Herein, by varying the Mg content in the ZnO lattice, we expect a colossal enhancement of the dielectric constant, which is beneficial for energy storage applications. The study covers a deep investigation of the transport properties, dielectric losses, dielectric dispersion, and modulus. The impedance spectroscopy, as a nondestructive and powerful method for electrical characterization, is used to study the transport changes in both grains and grain boundaries.

## 2. Experimental Procedure

### 2.1. Synthesis of ZnO:Mg NCs

The ZnO:Mg NCs preparation technique was provided in our earlier study [23]. Briefly, zinc acetate dehydrate “Zn(CH_3_COO)_2_. 2H_2_O; 0.02M” and magnesium acetate tetrahydrate “Mg (CH_3_COO)_2_. 4H_2_O”, with a purity of 99.99%, were used as starting materials. Citric acid (C_6_H_8_O_7_) was used as a stabilizer and deionized water was used as a solvent. The synthesis process could be resumed as follows: 400 mL of the solvent was used to dissolve the precursors. The mixture was stirred for 2 h at 50 °C and pre-heated at 80 °C until the solvent was completely removed. Then, we proceeded to the second thermal treatment at 350 °C for 3 h. The obtained powders were crushed and annealed at 500 °C for another 3 h to achieve the crystallized phase. The thermal process was performed in two steps in order to ensure well-organized crystalline phases and reduce the amount of impurity in each sample. The prepared powders are denominated as ZnO:Mg x% where *x* = 0, 1, 2, 3 and 5, representing the percentage of [Mg]/[Zn].The uniaxial press of 10 tons/cm^2^ was used to press the crystallized powders in order to obtain disk pellets of 1 cm diameter and a thickness “*e*” of 2 mm. The fabricated pellets were investigated during impedance measurements.

### 2.2. Experimental Study

XRD measurements were accomplished with a Philips X’Pert system (Malvern, UK), using a copper X-ray tube (λ = 1.54056 A°) at room temperature, at 40 kV and 100 mA. The obtained diffractometer in the angle domain was between 30° to 70° with a step size of 0.02°. The transmission electron microscopy (TEM) images were performed with a Philips CM30 microscope (Malvern, UK). The pellets were calcined and put among two platinum electrodes in a furnace. We proceeded to take the measurements by varying the temperature for each sample with a step of 10 °C from 200 to 260 °C. The study of the dielectric properties at different temperatures was conducted by gathering complex impedance data in the frequency domain between 40 Hz to 5 MHz with an impedance analyzer (Agilent 4294A, USA).

## 3. Results and Discussion

### 3.1. Structural and Morphological Studies

**Figure 1:**
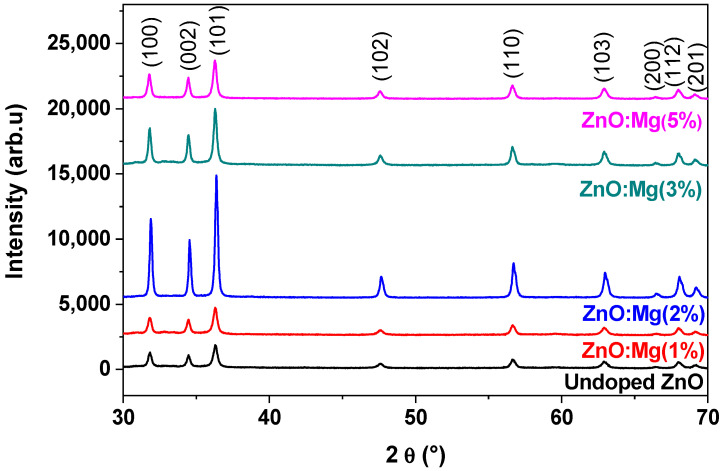
X-ray diffraction patterns of the prepared ZnO:Mg NCs.

Figure 1 represents the XRD patterns of pure and Mg-doped ZnO NCs. The obtained diffraction peaks highlighted the hexagonal wurtzite structure of ZnO of the different samples. The patterns demonstrate high crystalline quality along the (101) plane as a preferred orientation.

The ZnO structure remains unchanged after the Mg incorporation with low content. This affects the intensity and the wideness of the peaks. Indeed, the full width at half maximum of the peaks along the (101) plane decreased with Mg doping. The FWHM of the doped sample is reduced compared with that of a pure ZnO, highlighting the improvement of the crystalline structure. On other hand, the crystallite size increases with the incorporation of Mg, which was found to be greater than that of the undoped sample. Such an enhancement of the crystallite size is assigned to the Zn^2+^ substitution with Mg^2+^. The obtained values are gathered in Table 1.

**Figure 2:**
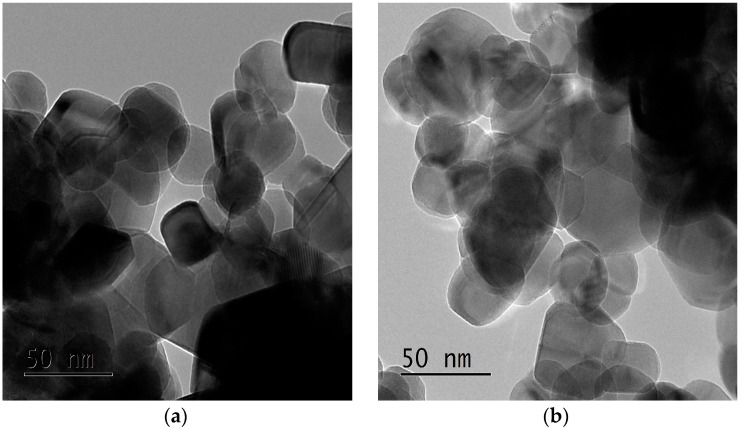
TEM images of undoped (**a**) and Mg3%-doped (**b**) ZnO NCs.

The TEM images of non-doped ZnO and ZnO:Mg (3%) NCs are given in Figure 2. A homogenous structure with a spherical shape could be observed on the fabricated NCs. The mean particle size is about 30 nm, which is in good agreement with the XRD analysis. Moreover, it is clear that the nanocrystal shape remains the same after doping. However, it is difficult to judge if the size decreases with Mg doping.

### 3.2. Impedance Spectroscopy

#### 3.2.1. Electrical Study

a. Impedance Spectra

**Figure 3:**
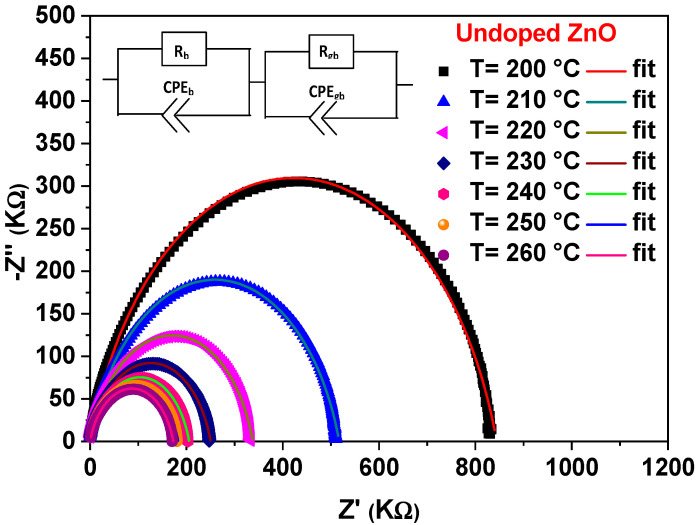
Nyquist plots of the undoped ZnO.

**Figure 4:**
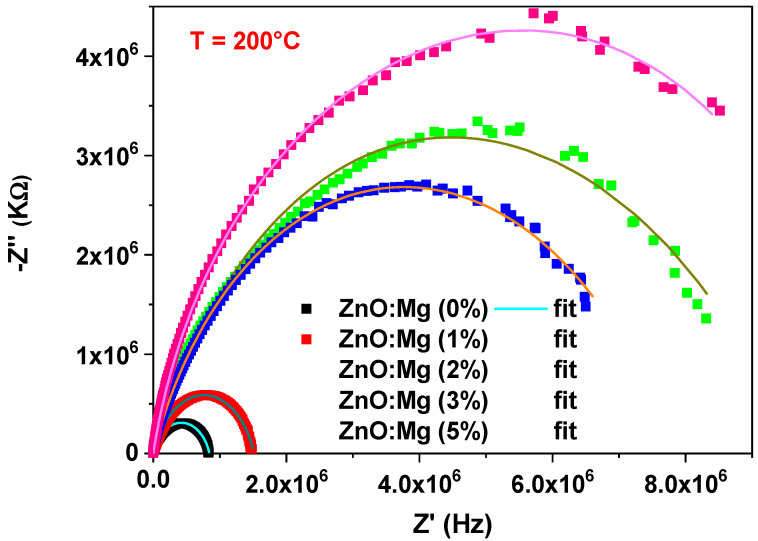
Experimental and theoretical Nyquist plots of ZnO:Mg NCs at T = 200 °C.

The complex impedance plots of undoped ZnO at various temperatures is given in Figure 3. We also studied the complex impedance changes at 200 °C for the various Mg percentages (see, Figure 4). Both figures illustrate the well-defined semicircles in the complex plane. This confirms the homogeneity and the single phase of the prepared doped samples. The centers of the semicircles are localized below the Z′ axis, which may be related to a non-Debye relaxation nature [30,31]. Compared with pure ZnO, the semicircles of Mg-doped ZnO NCs appeared at higher temperatures. Here, the increase in the Mg concentration enlarges the semicircle radius. However, the sample doped with 3% of Mg displays an opposite tendency. The diminution in the radius of the semicircle illustrates the enhanced of conductivity in the sample [32].

The investigation of the impedance plot requires modeling of the electric impedance with a corresponding circuit including one block consisting of a parallel association of the bulk resistance R and a capacitor.

In this case, the capacitor could be substituted by a constant phase element (CPE) *Z_CPE_* because of the semicircles depression [33]. *Z_CPE_* is given by:

(1) $$Z_{CPE}=\frac{1}{A_{0}{\left(j\omega \right)}^{n}}$$

Here, *j* denotes the imaginary unit, *ω* corresponds to the angular frequency, *A*_0_ is the capacitance value of *Z_CPE_*, and *n* is non-dimensional parameter (0 ≤ *n* ≤ 1) reflecting the trend to an exact semicircle [34].

The total impedance Z^*^ is described as follows:

*Z* = *Z*′ − *jZ*″ (2)

where

(3) $$Z^{\prime }=\frac{R\times \left(1+R\times A_{0}\times {\left(2\pi x\right)}^{n}\times \cos\left(\frac{\pi }{2}n\right)\right)}{1+2\times R\times A_{0}\times {\left(2\pi x\right)}^{n}\times \cos\left(\frac{\pi }{2}n\right)+{\left(R\times A_{0}\times {\left(2\pi x\right)}^{n}\right)}^{2}}$$

(4) $$-Z^{\prime \prime }=\frac{R^{2}\times A_{0}\times {\left(2\pi x\right)}^{n}\times \sin\left(\frac{\pi }{2\;}n\right)}{1+2\times R\times A_{0}\times {\left(2\pi x\right)}^{n}\times \cos\left(\frac{\pi }{2}n\right)+{\left(R\times A_{0}\times {\left(2\pi x\right)}^{n}\right)}^{2}}$$

The suggested model is used to fit the Nyquist plots, proving the viability between the equivalent circuit and the existing system (see Figure 3 and Figure 4). Table 1 lists the extract parameters. The capacitances, A_g_, is sized up at 10^−10^ F cm^−2^ S^n−1^, demonstrating that the grain boundary contribution is formed as a general response of the system [35,36]. The slight difference in ionic radius between Zn and Mg can generate a small displacement in the ZnO structure, since the Mg substitutes the interstitial Zn sites [23]. The n_g_ exponent values remain unaffected with increasing the Mg content oppositely to the resistance R_g_ that raises in this case (see, Table 2). This may be explained by the structural defects related to the doping. A similar behavior was observed for ZnO-doped Sb [37], where the enhancement of the resistance is coming from the substitution of the Zn^2+^ ions with Sb^3+^. Previous work by M. Ben Ali et al. [38] examining the Ni-doping influence on the electrical properties of ZTO assigned the increase in resistivity rate to the placement of the excess of the Ni-doping element on the grain boundaries. This demonstrates that Ni atoms are non-localized in the ZTO matrix, where the Ni excess leads to the formation of a Schottky barrier blocking the carrier’s transport. Based on these results, we can conclude that for high-doping concentrations, Mg atoms prefer to sit in the grain boundaries and form a barrier to hinder the carrier’s transport.

b. DC Conductivity

In order to inspect the electrical properties (DC conductivity, *σ_DC_*) of the Mg-doped ZnO NCs, we use the following expression:

(5) $$\sigma_{DC}=\frac{e}{SZ_{0}}$$

where *e* is the sample thickness, *S* represents the surface region, and $Z_{0}$ characterizes the resistance found based on the interception of the semicircles with the real axis.

**Figure 5:**
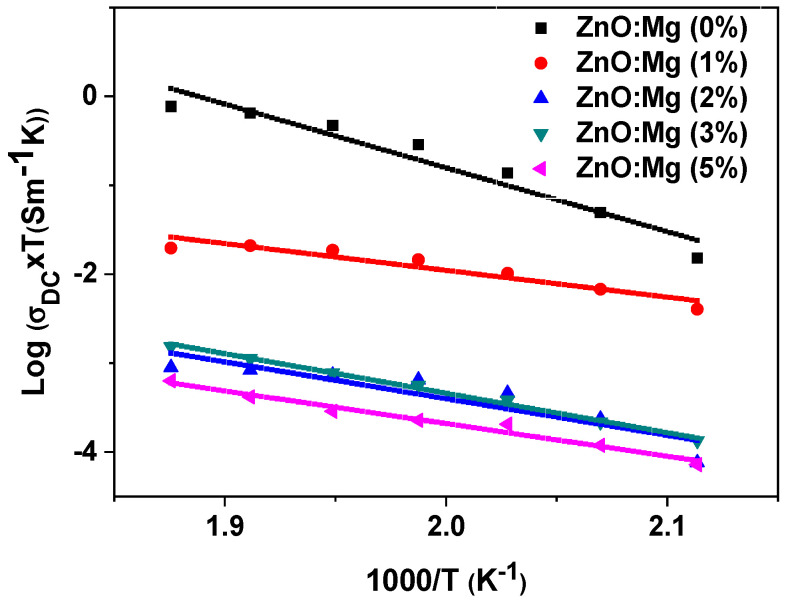
Semi-logarithmic plots of (σ_DC_T) versus 1000/T according to Arrhenius law.

The reciprocal temperature dependence to the DC conductivity is drawn in straight lines in Figure 5. Here, the Arrhenius law could be used:

(6) $$\sigma_{DC}T=\sigma_{0\;}\;.\exp\left(-\frac{E_{a}}{K_{B}.T}\right)$$

where, *K_B_* designed the Boltzmann constant and *E_a_* is the activation energy corresponding to the energy difference between the conduction levels and the donor. The $E_{a}$ values are deduced from the linear fit slope of the logarithmic DC conductivity $(\sigma_{dc}.T)\;$ versus $\frac{1000}{T}$. We found low activation energy values of doped samples in comparison with unmodified ZnO (see Table 3). The activation energy is estimated at 0.25 eV for the 1% Mg-doping concentration, which is ascribed to Zn^2+^ [39]. Besides, such an activation energy is found in the range (0.32 to 0.38 eV) for higher Mg percentages. These values originate from the oxygen vacancies V_O_ [40]. Chaari et al. [19] reported comparable results for ZnO ceramics doped with high concentrations of Sn_2_O_3_. The lower activation energy values reveal the source of the conduction phenomenon process in ZnO:Mg NCs, which is associated with the polaron hopping.

The DC conductivity decreases significantly with increasing the Mg doping amount due to the diminishing of the free electrons density. In fact, the imperfections act as trapping/scattering centers to reduce the number of free electrons. Further, the Mg atoms located at the grain boundaries behave as an electrical barrier that raise the carrier scattering, therefore, decreasing the conductivity [19,41].

c. AC conductivity

**Figure 6:**
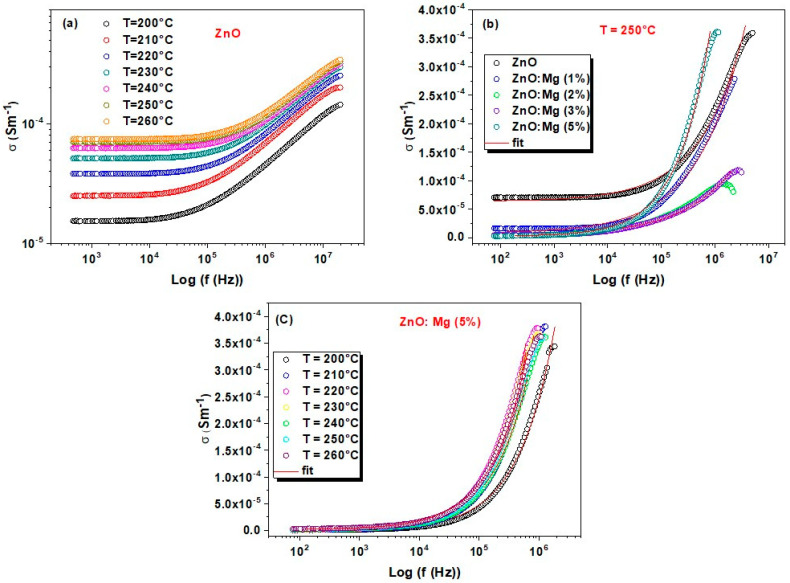
Frequency dependance of $\sigma_{AC\;}$ for: (**a**) undoped ZnO at different temperatures (**b**) all the ZnO:Mg samples at T = 250 °C and (**c**) ZnMg5 at different temperatures.

Figure 6a represents the frequency–temperature dependency of the AC conductivity. This is demonstrated by the Jonsher’s relationship [38,42]:

(7) $$\sigma_{AC\;}\left(\omega \right)=\sigma_{dc}+A\omega^{s}$$

where, $\;\sigma_{dc}$ is the DC conductivity associated to the flat region, $A\omega^{s}$ is the frequency reliant expression that characterizes the dispersion phenomenon, *A* a constant associated with the strength of polarizability [42], and *s* is a factor verifying 0≤s≤1  [43] which describes the interaction level between the mobile ions and the matrix. All these parameters depend on temperature [44].

As we can see from Figure 6a, all the curves depend on temperature. The power law is obeyed for the different ZnO:Mg samples since the fit matches well with the experimental data in Figure 6a–c. At low-frequency range, the plots show an unaffected flat region corresponding to DC conductivity, whereas the dispersion at high-frequency range is related to AC conductivity. Ionic conductors are characterized with such a behavior [45]. For the lower frequencies, the conductivity is reduced with further Mg addition due to the placement of the doping element on the interstitial position in the ZnO lattice that enhances structural defects concentration (Figure 6b). At the high-frequency region, the conductivity continues to decrease from 1 to 3% of Mg content while it shows an increase for 5% of Mg doping. These latest samples display a high conductivity due to the increase in oxygen vacancies that lead to augmentation of the hopping charge carrier’s concentration [46].

**Figure 7:**
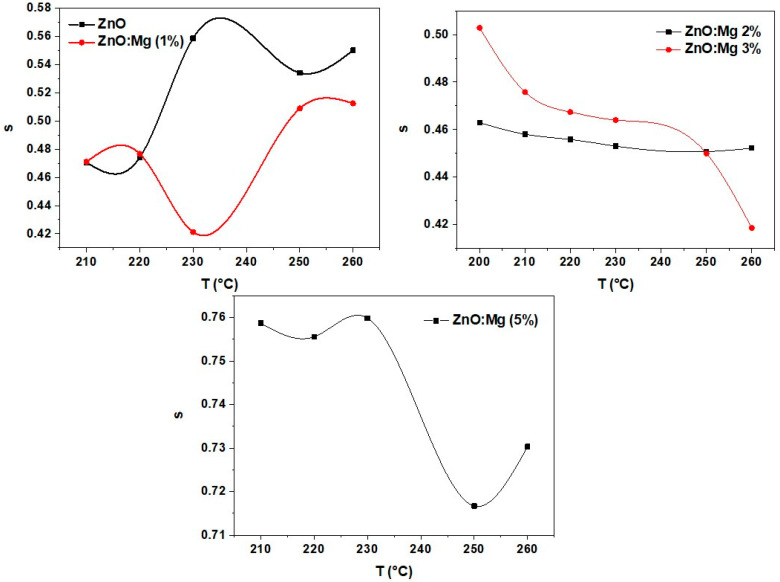
Temperature dependence of the parameter s for all the ZnO:Mg samples.

The theoretical fits give $\sigma_{\mathrm{dc}}$ and *s* values which can explain the mechanism related to the conduction process. The *s* parameter is dependent on the ZnO and ZnO:Mg (1%) temperature, shown in Figure 7. It slightly increases from 0.42 to 0.56 with temperature. This increase could be attributed to the quantum mechanical tunneling (QMT) conduction model [47]. Here, s is expressed as [48]:

(8) $$s=1-\frac{4}{\ln\left(\frac{1}{\omega \tau_{0}}\right)-\frac{W_{H}}{K_{B}T}}$$

where, $K_{B}$ is the Boltzmann constant, $W_{H}$ is the energy of the polaron hopping, and $\tau_{0}$ is a typical relaxation time. When $W_{H}$ *>>* $K_{B}T$, the expression can be simplified as follows:

(9) $$s=1+\frac{4k_{B}T}{W_{H}}$$

For the samples ZnM2 and ZnMg3, the exponent *s* was found to vary from 0.50 to 0.42 with increasing temperature from 200 to 260 °C. However, its value is more important for the ZnO:Mg (5%) sample, around 0.7, and decreases slightly with temperature. The diminution of the *s* parameter leads to the principal transfer route which is correlated with the barrier hopping (CBH) model [49,50,51], where charge carriers skip among sites over the potential barrier parting them instead of tunneling via the barrier [52]. The parameter *s* obeys the following equation [53]:

(10) $$s=1-\frac{6K_{B}T}{W_{M}}$$

$W_{M}$ is the upper barrier height at an infinite separation. This is also identified as the energy relative to polaron binding in its localized sites.

**Figure 8:**
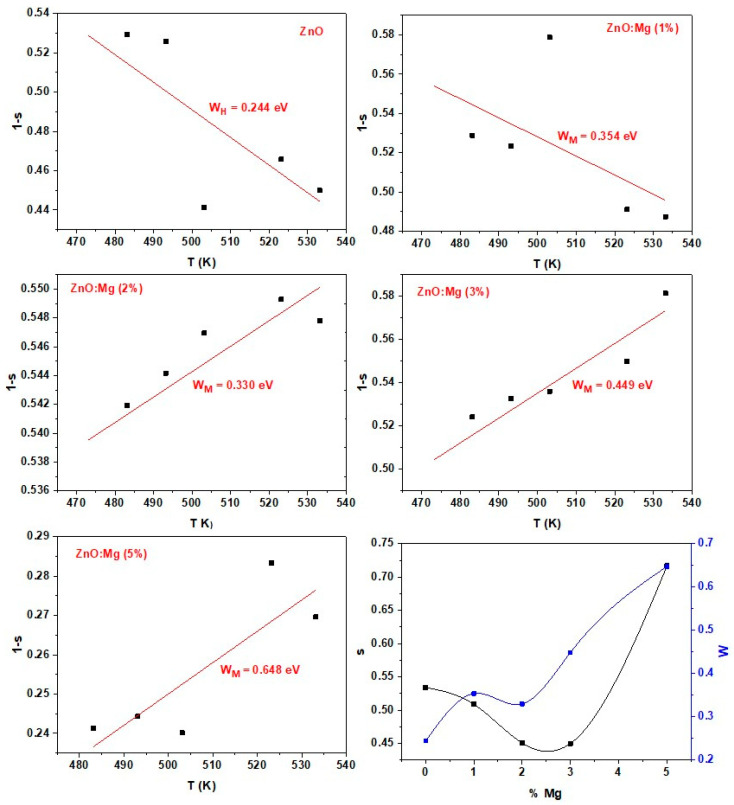
Variations of the parameters (1-s), and W_M_ with temperature, and %Mg, respectively.

Figure 8 displays the (1-s) plots versus the temperature that gives the $W_{M}\;\mathrm{values}\;$. *W_M_* and ***s*** raised with Mg concentration (see Figure 8). Such a change in conduction model is assigned to the enhancement of the charge carrier induced by more Mg^2+^ ions in the ZnO lattice.

#### 3.2.2. Dielectric Study

a. Permittivity and loss studies

The complex dielectric constants describe the dielectric properties of materials. They are determined from *Z*′ and *Z*″ (Equations (3) and (4), respectively) and given by the following formula [38]:

(11) $$\varepsilon \left(\omega \right)=\varepsilon^{\prime }\left(\omega \right)-j\varepsilon^{\prime \prime }\left(\omega \right)=\frac{1}{j\omega C_{0}\left(Z^{\prime }+jZ^{\prime \prime }\right)}\;$$

where the real *ε*′ and the imaginary *ε*″ parts illustrate the quantities of energy accumulated and dispersed in the dielectric owing to the applied electric field, correspondingly. Their expressions are:

(12) $$\varepsilon \prime \left(\omega \right)=-\frac{Z^{\prime \prime }}{\omega C_{0}\left({Z^{\prime }}^{2}+{Z^{\prime \prime }}^{2}\right)}\;$$

(13) $$\varepsilon^{\prime \prime }\left(\omega \right)=\frac{Z^{\prime }}{\omega C_{0}\left({Z^{\prime }}^{2}+{Z^{\prime \prime }}^{2}\right)}\;$$

Here, $C_{0}$ is geometrical capacitance of samples ($C_{0}=\frac{\varepsilon_{0}S}{e}$, *ε*_0_ is the permittivity of the vacuum, S is the cross-sectional area of the flat surface of the pellet, and e is the thickness). *Z*′ and *Z*″ are the real and the imaginary parts of impedance. The dielectric loss (tan δ) is calculated from the values of the dielectric constants and could be written as follows [54]:

(14) $$\tan\;\delta =\frac{\varepsilon^{\prime \prime }}{\varepsilon^{\prime }}$$

**Figure 9:**
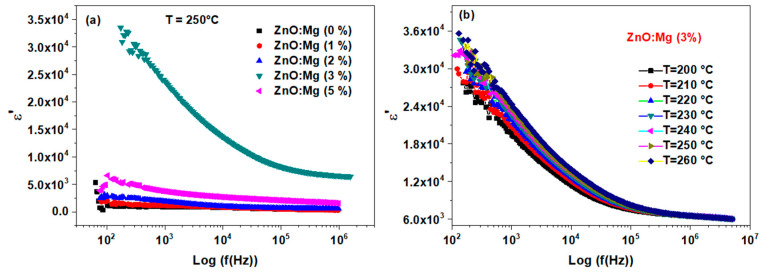
Dielectric constant *ε*′ versus frequency for (**a**) different Mg doping and (**b**) ZnO:Mg (3%) sample, at various temperatures.

Figure 9a depicts the frequency dispersion *ε*′ for all the ZnO:Mg samples. It shows an increase of *ε*′ with the Mg content. For each sample, the dielectric constant gradually reduces as the frequency augments, and reaches an almost constant value (relatively high) in the high frequencies range.

Figure 9b depicts the frequency dispersion *ε*′ versus the temperature of the ZnO:Mg (3%) sample. As shown, *ε*′ is gradually reduced through the increasing of the frequency to reach almost a constant value in the high frequencies range. This way points to a Debye-type dielectric dispersion [55,56]. According to the Maxwell–Wagner model [57], the large values of *ε*′ is related to the large polarization effect produced at the grain boundaries due to the charge carrier’s migration between grain boundaries under the effect of the applied external field [58]. At high frequencies range, the decrease in *ε*′ values could be explained by polarizability loss, since the dipoles are enabled to rotate quickly which leads to a delay among frequencies of the oscillating dipole and applied fields [59]. It can be pointed out that the reached *ε*′ value (at medium and high frequencies) is considered relatively high, making the ZnO:Mg samples very promising for energy storage applications.

The imaginary part *ε*″ also decreases with the frequency but it varies slightly versus the temperature. This demonstrates the loss of polarization with the frequency by the disappearance of dipoles or to their inability to rotate properly at high frequencies [60].

**Figure 10:**
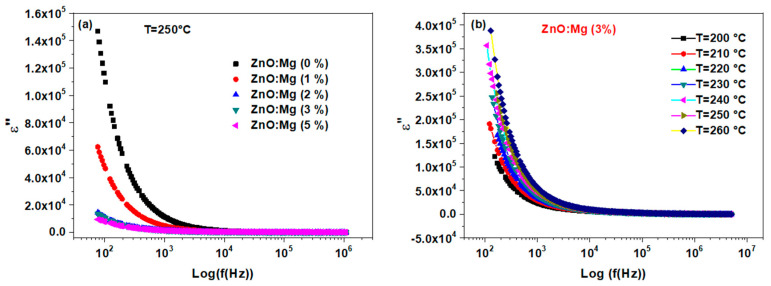
Frequency dependence curves of the imaginary part $\varepsilon^{\prime \prime }$ (**a**) for the ZnO:Mg (3%) sample at different temperatures and (**b**) for different Mg content.

**Figure 11:**
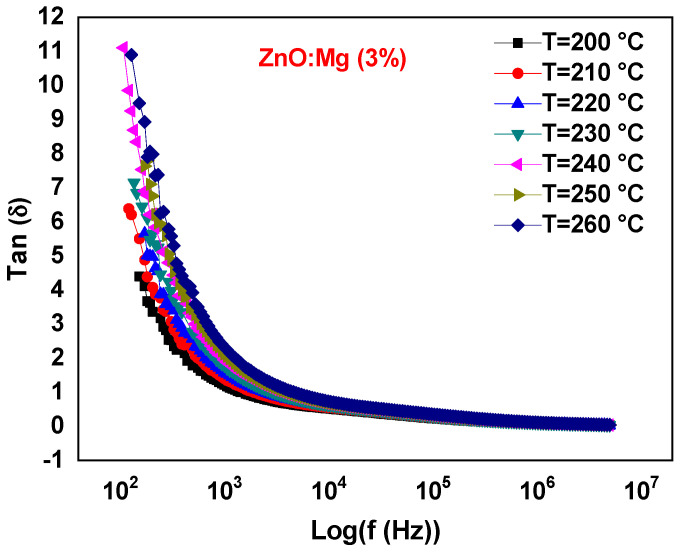
Dependance of the dielectric loss tan *δ* on the frequency for the sample ZnO:Mg 3% at different temperatures.

We located a considerable reduction of the imaginary part *ε*″ with the rate of the Mg doping which confirms the rule of the space charge [61] (see Figure 10a). The same performance was also monitored by Hafef et al. [24] with high MgO-doping rates (10% and 20% of MgO). This also was explained by the separation of the MgO phase in the ZnO host matrix. In this work, the Mg is well incorporated into the ZnO lattice and hence, it induces less crystallinity, as was previously shown in the XRD analysis. Thus, this behavior could be related to the increased defects density induced by further Mg incorporation, which leads to less dielectric polarization. Moreover, *ε*″ increases with the temperature (see Figure 10b) where no peaks in the loss tangent curves are observed (Figure 11). This demonstrates the slightly high conduction losses [62]. Finally, the present material could be an encouraging candidate for device applications acting in the high-frequency range.

b. Modulus analysis

The electric modulus is generally used to examine the electrical characteristics of bulk materials. At low frequencies, the conductivity phenomenon usually hides the interfacial polarization generally present in such materials [63]. To surmount this difficulty, modulus is mostly appropriate to obtain phenomena such as the conductivity relaxation times and the electrode polarization. The complex electric modulus is written as [64]:

(15) $$M^{*}=\frac{1}{\varepsilon^{*}\left(\omega \right)}=M^{\prime }\left(\omega \right)+jM^{\prime \prime }\left(\omega \right)=j\omega C_{0}Z^{*}$$

where, $M^{\prime }=\omega C_{0}Z^{\prime \prime }$ and $M^{\prime \prime }=\omega C_{0}Z^{\prime }$ are the real and imaginary parts of the complex electric modulus, respectively.

**Figure 12:**
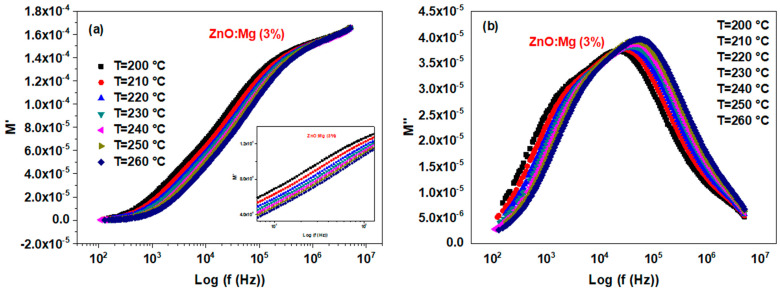
Plots of (**a**) *M*′ and (**b**) *M*″ within frequency for various temperatures for the ZnO:Mg (3%) sample.

Figure 12a represents the variations of *M*′ within the frequency at different temperatures. It raises with increasing frequency to reach a flat zone to the highest frequency values corresponding to the limiting value of *M*′. This behavior illustrates the weak contribution of the electrode polarization that could be neglected for Mg-doped ZnO NCs [65]. Such behavior was also detected for Na-doped ZnO NCs [22].

The frequency reliance of *M*″ with the temperature for ZnO:Mg (3%) is represented in Figure 12b. The ${M^{\prime \prime }}_{\max}$ position shifts near the high-frequency region when the temperature increases, and the shift of the ${M^{\prime \prime }}_{\max}$ position is usually related to the conductivity relaxation. An asymmetric peak could be observed for each investigated temperature, which exhibited the non-Debye kind of behavior in the relaxation of the Mg-doped ZnO NCs [66].

The detected peak aids in determining the relaxation time τ using the following relationship [58]:

(16) $$\tau =\frac{1}{\omega_{max}}=\frac{1}{2\pi \;f_{max}}$$

where $f_{max}$ designates the relaxation frequency.

**Figure 13:**
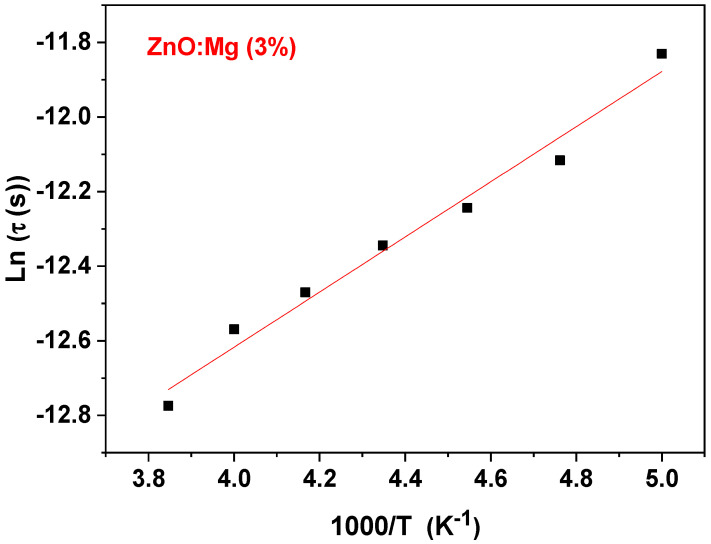
Plot of *Ln* τ versus 1000/T.

The change of ${M^{\prime \prime }}_{\max}$ with temperature images the enhancement of the dielectric relaxation time τ with temperature [37]. Such a change affirms the thermal stimulation of the dielectric relaxation [67]. As shown in Figure 13, Lnτ varies linearly with the inverse of the absolute temperature *T*, following the equation:

(17) $$\tau =\tau_{0}\exp\left(-\frac{E_{a}}{K_{B}T}\right)$$

where, *τ*_0_ is the pre-exponential factor. From a linear fit of the plot, the value of the activation energy $E_{a}$ is estimated to 0.73 eV, which is quite different to that determined while considering the plot of Ln(**σ**_dc_T). Hence, it provides information about the non-statistic distribution of the Mg^2+^ ions in the ZnO matrix and suggests an arbitrary conductivity. Therefore, the relaxation of dipoles manifests itself arbitrarily [37,68].

## 4. Conclusions

Mg-doped ZnO NCs were synthesized via sol-gel method. The electrical and dielectric characteristics were investigated as function of temperature and frequency, using impedance spectroscopy. The conductivity and the activation energy are influenced by Mg concentration. From temperature reliance, we reveal that the conduction mechanism is managed from the model of the hopping correlated barrier (CBH). Dielectric features are largely affected by Mg doping due to less dielectric polarization. The low dielectric losses at high frequencies and the shift toward high frequencies of $M^{\prime \prime }$ with temperature, make the Mg-doped ZnO NCs a suitable material for application in non-linear optics. The analysis of the electrical modulus illustrates an extension in the relaxation time with further Mg incorporation. All the dielectric properties relative to the prepared ZnO:Mg NCs are of interest to bring a considerable influence to several technological applications, such as microwave devices.

## Figures and Tables

**Table 1:** Structural parameters of pure ZnO and ZnO:Mg NCs [23].

	FWHM of (101) Peak (Rad)	D (nm)
ZnO:Mg 0%	0.0060	26.82
ZnO:Mg 1%	0.0054	30.47
ZnO:Mg 2%	0.0038	42.96
ZnO:Mg 3%	0.0044	36.75
ZnO:Mg 5%	0.0048	33.69

**Table 2:** Fitting parameters relative to the equivalent circuit elements for all the ZnO:Mg samples.

	R_b_ (10^5^ Ω)	A_g_ (10^−10^ F cm^−2^ S^n−1^)	n_g_
Mg 0%	1.82848	1.9535	0.8023
Mg 1%	8.17662	1.7426	0.8325
Mg 2%	35.72726	2.3201	0.7633
Mg 3%	30.83072	2.4610	0.7645
Mg 5%	49.20645	1.6784	0.8153

**Table 3:** $\sigma_{dc}$ and E_a_ changes with Mg content at T = 250 °C.

% Mg	0	1	2	3	5
**R_b_ (kΩ)**	840.56	1481.82	6589.22	8321.14	8424.57
$\sigma_{DC}$ **(10^−6^ S.m^−1^)**	15.15	8.56	1.93	1.53	1.51
***E_a_* (eV)**	0.62	0.25	0.36	0.38	0.32
